# Reassessing the Determinants of Breeding Synchrony in Ungulates

**DOI:** 10.1371/journal.pone.0041444

**Published:** 2012-07-20

**Authors:** Annie K. English, Aliénor L. M. Chauvenet, Kamran Safi, Nathalie Pettorelli

**Affiliations:** 1 Imperial College London, Division of Biology, Ascot, United Kingdom; 2 The Zoological Society of London, Institute of Zoology, London, United Kingdom; 3 Max Planck Institute for Ornithology, Radolfzell, Germany; Institut Pluridisciplinaire Hubert Curien, France

## Abstract

Predicting the consequences of climate change is a major challenge in ecology and wildlife management. While the impact of changes in climatic conditions on distribution ranges has been documented for many organisms, the consequences of changes in resource dynamics for species' overall performance have seldom been investigated. This study addresses this gap by identifying the factors shaping the reproductive synchrony of ungulates. In temporally-variable environments, reproductive phenology of individuals is a key determinant of fitness, with the timing of reproduction affecting their reproductive output and future performance. We used a satellite-based index of resource availability to explore how the level of seasonality and inter-annual variability in resource dynamics affect birth season length of ungulate populations. Contrary to what was previously thought, we found that both the degree of seasonal fluctuation in resource dynamics and inter-annual changes in resource availability influence the degree of birth synchrony within wild ungulate populations. Our results highlight how conclusions from previous interspecific analyses, which did not consider the existence of shared life-history among species, should be treated with caution. They also support the existence of a multi-faceted link between temporal variation in resource availability and breeding synchrony in terrestrial mammals, and increase our understanding of the mechanisms shaping reproductive synchrony in large herbivores, thus enhancing our ability to predict the potential impacts of climate change on biodiversity.

## Introduction

Understanding how climate influences vertebrate populations and projecting the future ecological consequences of climatic change are major challenges in ecology and wildlife management [1], [2]. In just a century, global surface temperature has increased by 0.74±0.18°C [3], leading to melting of glaciers, reduction in surface sea ice and increases in sea level [4]. Climate change is typically considered in terms of changes in average conditions, but these will be far from the only expected changes: shifts in seasonal patterns and increased occurrence of extreme climatic events are also expected to occur [3], [5]. Changes in seasonal patterns have already been reported in Europe, Africa and America [3]; such changes can disrupt trophic interactions between predator and prey [6], [7] which can be particularly strong in seasonal environments [8]. Interestingly, climate change research has mostly focused on how changes in the timing of vegetation phenology might affect demographic rates and distribution range [7], [9]–[11], yet little is known regarding the potential effects of such changes on behaviour and reproductive phenology [12], [13].

In temporally variable environments, reproductive phenology is a key determinant of fitness with the timing of reproduction being sometimes reported to strongly affect immediate reproductive output (e.g., annual reproductive success) and future performance of animals (e.g., lifetime reproductive success; [14]–[17]). At the individual level, various factors such as photoperiod, vegetation onset, or body condition of the mother correlate with the timing of parturition [18]–[21]. At the population level, parturitions can be more or less synchronous: for example, up to 64% of banded mongoose (*Mungos mungo*) females can give birth in one night [22]; the entire spawning period for Japanese Charr (*Salvelinus leucomaenis japonicas*) can take up just 11 days [23]. Nevertheless, there can be a large variation in the length of the birth season within species and within populations [24]–[27]. Various hypotheses have been proposed to explain the reported high level of variability in the level of birth synchrony. The most supported one is that resource availability dynamics is the primary driver of birth season length, with species exhibiting a high level of birth synchrony generally found in highly seasonal habitats [24]. However, other ecological processes, such as predation avoidance, have also been hypothesized to shape the level of reproductive synchrony [28]–[30].

The aim of this study is to identify the factors shaping birth season length (Table 1) in terrestrial mammals. We decided to focus on ungulates, one of the most diverse land dwelling groups of vertebrates on Earth [31]. Knowing which factors influence birth season length in this group, and therefore the level of breeding synchrony, could be fundamental to understanding which populations are at risk of decline due to mismatch with their changing environment [7], [32]). Since Rutberg's [24] review on this issue, a large amount of new data has been collected and published. Global measures of primary productivity have, moreover, become available [33], as well as refinements in statistical analyses. In particular, a potential flaw associated with the analyses performed by Rutberg [24] is linked to the assumption of independence with regards to the interspecific data [34], [35], and methods are now available to correct for phylogenetic non-independence [36]–[43].

**Table 1:** The variables hypothesized to influence birth season length in ungulate populations, with the rationale behind their inclusion.

Variable	Hypothesis	References
Latitude	Populations inhabiting higher latitudes should have a shorter birth season	[24], [88]
Seasonality (Contingency)	Populations inhabiting more seasonal environments should have a shorter birth season, due to the window of optimal resource availability being shorter in these locations.	[8]
Inter-annual variability (Constancy)	In less constant environments there is a longer time window during which optimal vegetation conditions could occur; therefore the birth season would be longer than in more constant environments.	[26], [59]
Diet type	As grass is more seasonal than browse, grazers are expected to have a shorter birth season length than mixed feeders or browsers.	[24], [26]
Calf Behaviour	Populations with following young should have a shorter birth season than populations with hiding young.	[24], [27], [82]
Gregariousness	Predation avoidance will be maximized for populations with following young that are aggregated in large herds, as this can cause predator confusion, saturation and defense. Gregarious populations should have a shorter birth season length than solitary species.	[27]

## Materials and Methods

### Ungulate data

A thorough literature search using ISI Web of Knowledge™ (http://apps.isiknowledge.com) was conducted during April and May 2011. We used the keywords ‘parturition’, ‘birth’, ‘season’, combined first with ‘ungulate’, and then with individual ungulate species' common and scientific names. Parturition dates or birth seasons for individual ungulate populations were only collected from published field studies. There was a large variation in the sample sizes and details reported, with, e.g., study dates ranging from 1960 to 2006 and study length from one to 30 years.

Methods that relied on birth estimates from fetal measurements were discarded: variation in gestation length and calf birth weight has been reported for many species [18], [44]–[46], and thus these methods are inaccurate for predicting birth dates. However, we did consider studies relying on birth estimates from fetal measurements for populations where pregnant females were found throughout the year, as these studies adequately demonstrate asynchrony.

**Figure 1:**
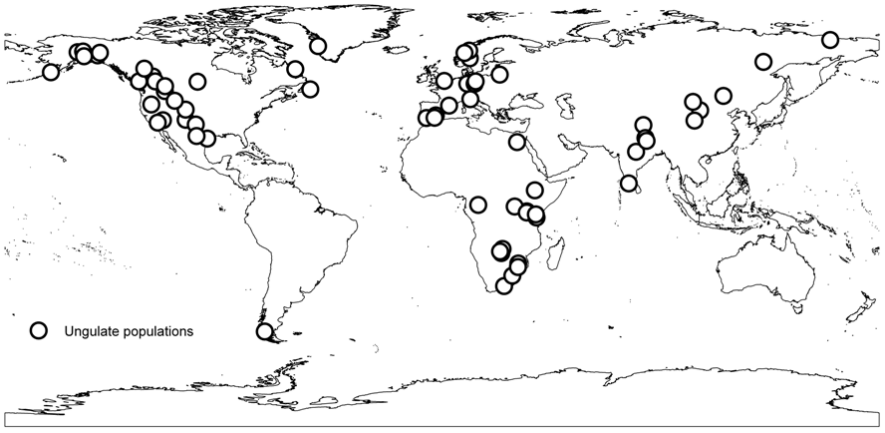
Distribution of the 70 ungulate populations used in the analysis.

There is no standard way to report information about birth synchrony across studies, and whilst compiling the dataset we found that most studies attempted to infer the whole birth season length for the entire population. The second most popular metric was the number of days in which 80% of the births occurred, but this information was only available for 50% of the populations. To maximize sample size, we therefore decided to only consider studies that inferred the whole birth season length for the entire population and from wild or semi-wild populations. The ‘birth season length’ was recorded as the number of days from the first birth until the last birth in the population in a given year. For studies that recorded births in longer time units (e.g., weeks or months), the midpoint of the unit was used to calculate the birth season length in days. Data was obtained for 70 different ungulate populations, and included 38 species ranging 65 separate locations (Figure 1). Five locations were associated with information for two species.

Species characteristics (calf behaviour, diet type, and level of gregariousness) were obtained from Walker [47], Kingdon [48], Huffman [49] and Wilson and Mittermeier [50]. Calf behaviour was defined as either ‘hider’ or ‘follower’ depending on whether neonates lay concealed for the first few days after birth or followed their mother. Diet type was classified as ‘grazer’, ‘mixed feeder’ or ‘browser’ [27], [51], [52], and the level of gregariousness was described using an ordinal scale; ‘1’ was assigned to solitary species, ‘2’ for species found in groups of less than 10 individuals, ‘3’ for groups of 11 to 100, ‘4’ if the species is found in herds of 100 s, and ‘5’ for herds of 1000 s.

### Normalized Difference Vegetation Index

ArcInfo 9.3.1 [53] was used to create a polygon for each of the 65 locations described in the literature. If the location given was listed in the World Database of Protected Areas, the area from this database [54] was used. Where the location name matched that of a protected area in the same country, or where the name was dissimilar, but the description and coordinates matched those of a protected area then the described location was taken to be the same as the protected area. For any study area which spanned more than one protected area, the protected areas were combined. Administrative regions were extracted from the global database of administrative areas (available at www.gadm.org), and islands were extracted from the natural earth database (www.naturalearthdata.com). For the locations with no official boundary, an area was created by taking the published coordinates as the centre point of a circle with an area of that quoted in the paper. When no area was quoted, the area taken was based on the level of detail of the coordinates. If the coordinates were to the nearest degree, an area of 5000 km^2^ was used, and where minutes were given, an area of 500 km^2^ was assigned.

Resource availability was indexed using the Normalized Difference Vegetation index (NDVI; [33], [55]). Pixels containing NDVI data that were located within each of these areas were then extracted from the Global Inventory Modelling and Mapping Studies dataset [56], which contains bi-monthly values at a resolution of 64 km^2^. From this dataset pixels from 1982 to 2008 were used. If no pixel was located directly within the polygon, the closest pixel(s) were used. These pixels were ‘smoothed’ to correct for common anomalies that occur in the data due to interference in the reflectance to the satellite caused by weather conditions, pollution, water and snow cover [33]. Smoothing was conducted based on the method described in Garonna and colleagues [57]. Pixels located in water were removed from the dataset. Any pixel that had three or more consecutive negative values was also removed. Rapid changes (of more than 0.25) followed by a rapid return to the original values were identified, assumed to be anomalies, and therefore replaced by the average of the previous and following values. If two anomalies occurred consecutively then they were replaced with a weighted average. For areas experiencing snow cover for part of the year, it is expected that NDVI values measured during the winter months of each year will be negative (and therefore are not anomalies). For the most northerly locations (Arctic Slope, Caribou River, Denali National Park, Hungry Horse Reservoir, Mackenzie Valley, Nordenskioldland, Ram Mountain and West Greenland), negative NDVI values recorded in the winter season were manually replaced with zeros before smoothing. The winter season was taken to be a maximum of a 6 month period between November and May, but varied in time and length depending on the pixel and the year. If any location had less than 50% of the pixels remaining it was excluded from the analysis. As a final stage, the average NDVI values across pixels and across years were calculated for each location.

Our aim was to characterize the level of “predictability” in vegetation dynamics (as indexed using the NDVI) over the years 1982–2008 (which represents the longest NDVI time series we were able to access) in the areas where the birth season length data were collected. To fulfill this aim, the level of seasonality (contingency sensu Colwell [58]) and inter-annual variability (constancy sensu Colwell [58]) in vegetation dynamics for each location were calculated using the method described in Colwell [58] (see [59] for a recent application). Within this framework, NDVI values are discretized and predictability is understood as the sum of constancy and contingency. Discretization of the NDVI data can entail a loss of information: because our analysis is carried out at the global scale, this loss is expected to have a low impact on our results. Constancy is here defined as a parameter assessing the importance of year-to-year stochastic variation (e.g., the higher the NDVI constancy, the lower the level of inter-annual variability in NDVI dynamics), while contingency measures the level of seasonality in the “average” annual pattern (and thus reflects how strong seasonality is in a given area; see Text S1). Constancy and contingency both vary between 0 and 1.

### Statistical Analysis

Because there were only 5 locations where birth season length was available for more than one species, we hypothesized that non-independence of data due to shared evolutionary history would be the main source of bias when exploring links between birth season length, environmental factors and species characteristics. Therefore, the non-independence of data due to shared evolutionary history was corrected for by incorporating phylogenetic information into the analysis [41]. To incorporate this phylogenetic information, a phylogenetic tree of the species included in this study was extracted from the species level supertree of Bininda-Emonds and colleagues [60]. Populations were then added to this tree by splitting the tips of the tree to create polytomies with branch lengths of ∼0. The package ‘geiger’ [61] was used in R (http://www.R-project.org) to determine which evolutionary model (if any) best described the evolution of birth season length across the phylogeny [34], [35], [62]. Akaike's Information Criterion (AIC) was used to determine which model best fitted the data [63]. Transformation of the phylogeny by the Maximum Likelihood of Pagel's lambda [64] gave the best description of birth season length evolution.

By integrating the model best describing the evolution of birth season length across the given phylogeny, we were able to statistically take into account the non-independence of the data due to shared ancestry to explore the effects of inter-annual variability in vegetation dynamics (constancy) and level of seasonality (contingency) along with the effects of latitude, diet type, level of gregariousness and calf behaviour on the log of birth season length (in days). There are currently a number of phylogenetic comparative methods available [65]. We used Phylogenetic Generalized Least Squares (PGLS) methods, which are regarded as the most general and robust way of correcting for phylogenetic non-independence in data [34], [41], [65]. Models were compared using AIC corrected for small sample sizes (AICc; [66]). Spatial autocorrelation, where populations from nearby locations are not independent of each other due to shared environment can potentially bias model estimates. Moran's I was assessed with Moran's I standard deviate [67] in R (package ‘spdep’; [68]). We checked both the original dataset on the log of birth season length and the residuals from the best model. No significant spatial autocorrelation was found in the original dataset or in the residuals from the best model for birth synchrony, and therefore spatial autocorrelation was not corrected for.

## Results and Discussion

**Figure 2:**
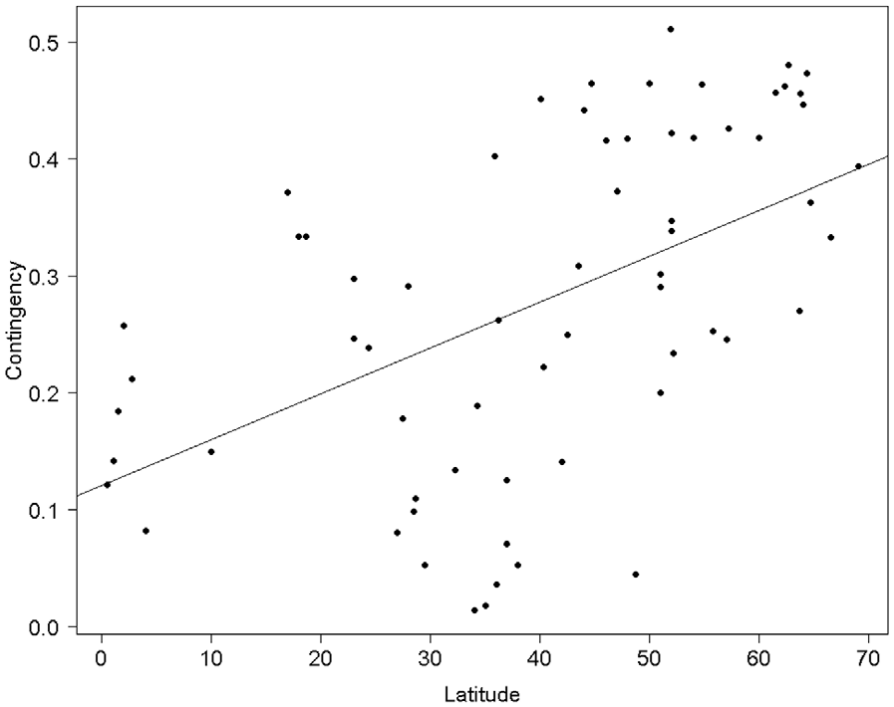
Correlation between latitude and NDVI contingency (NDVI contingency refers to a measure of seasonal variation in vegetation dynamics, as assessed from NDVI time-series).

**Figure 3:**
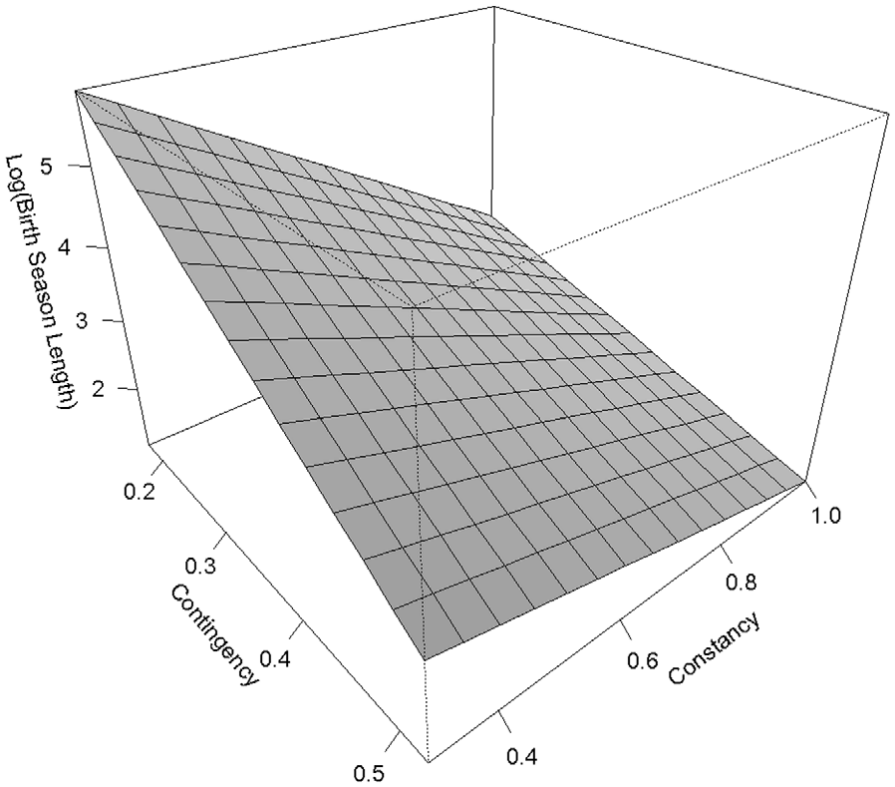
Expected changes in birth season length (according to the best model for birth season length) with changes in NDVI constancy (i.e., our measure of inter-annual variability in NDVI dynamics) and NDVI contingency (i.e., our measure of the strength of the seasonal pattern in NDVI dynamics). In this figure, birth season length is on the log scale. Within our dataset (used to generate this figure), contingency ranges from 0 to 0.5 and constancy from 0 to 1.

Birth season length ranged from 10 to 365 days, with a mean of 127.47±131.72 days (n = 70). As expected, latitude had an influence on the level of seasonality in vegetation dynamics (contingency): latitude explained 27.16% of the level of seasonality in NDVI dynamics (slope = 71.70±13.87, t = 5.17, P<0.001; Figure 2) in the 65 locations considered. The level of inter-annual variation in NDVI dynamics (constancy), however, was not correlated with latitude (slope = −11.17±12.25, t = −0.91, P = 0.36). NDVI constancy and contingency were significantly negatively correlated (Pearson's correlation test: t = −11.69, P<0.001, R^2^ = 0.66): increased degree of seasonality in NDVI dynamics was therefore associated with decreasing levels of inter-annual variability in NDVI dynamics. In accordance with Rutberg's previous results, birth season length was shorter for populations at higher latitudes (slope = −0.026±0.007, t = −3.94, P<0.001; [24]). Our analyses, however, revealed that considering both constancy and contingency, as opposed to latitude, best fitted the observed variation in ungulate birth season length (Tables 2 and 3; Figure 3, Figure S1).

**Table 2:** Models considered while modeling birth season length.

Model	AICc
Contingency * Constancy	165.80
Contingency * Constancy + Gregariousness	166.48
Contingency + Constancy	168.02
Contingency * Constancy + Calf Behaviour	168.05
Contingency + Constancy + Gregariousness	168.66
Contingency + Constancy + Latitude	169.49
Contingency * Constancy + Diet	169.88
Contingency + Constancy + Calf Behaviour	170.2
Contingency + Constancy + Diet	172.13
Latitude + Contingency	185.28
Latitude + Contingency + Calf Behaviour	186.77
Latitude + Contingency + Diet	189.50
Latitude + Contingency + Gregariousness	190.08
Latitude + Contingency * Diet	193.86
Latitude	194.41
Contingency	194.53
Latitude + Constancy	195.42
Latitude + Diet	198.76
Latitude + Constancy + Diet	199.88
Latitude + Constancy * Diet	203.75
Constancy	212.33
Calf Behaviour	212.99
Diet	214.23

Models are ranked according to their associated Akaike Information Criterion corrected for small sample sizes (AICc). “*” Indicates the presence of an interaction between the variables on both sides of the sign (e.g., “Contingency*Constancy” should be read as “Contingency + Constancy + Contingency x Constancy”). “Diet” refers to diet type, “Gregariousness” to the level of gregariousness.

**Table 3:** Estimates for the parameters associated to the model best fitting the birth season length data considered.

Parameter	Value	SE	t	P
Intercept	9.33	0.65	14.37	<0.001
Contingency	−11.53	1.57	−7.36	<0.001
Constancy	−6.18	1.00	−6.18	<0.001
Contingency*Constancy	7.67	3.67	2.09	0.04

Estimates (Value) are provided along their associated standard errors (SE) and associated statistics (t value, P value).

Finding the variation in ungulate birth season length to be better explained by the level of seasonality and inter-annual variability in NDVI dynamics rather than latitude provides new insight into the evolution of breeding synchrony. Most ungulates are indeed thought of as K strategists [69], [70]: they tend to be relatively large and long lived, prioritising the quality of offspring produced over quantity. This in turn leads to a longer reproductive cycle and places higher energetic requirements on the females during lactation [71], causing a fitness advantage for individuals who match the most energy demanding stage of reproduction to the optimum time for resource availability [8]. The timing of parturition is thus limited by the length of the other stages of the reproductive cycle. Ungulates have been found to vary their gestation length (see e.g. [45], [46], [72]–[74]) but it is likely that this can only be done to a certain degree. Our results thus support the long established resource availability hypothesis that suggests a link between seasonal variation in resource availability and breeding synchrony in terrestrial mammals [27], [75], [76] but add an important insight. Both the levels of seasonality and inter-annual variability in resource dynamics shaped the level of breeding synchrony in ungulates across the globe.

Some limitations exist regarding the reported relationship between NDVI dynamics and ungulate breeding synchrony. NDVI integrates the composition of species within the plant community, the vegetation form, vigor, and structure, the vegetation density in vertical and horizontal directions, the reflection, absorption, and transmission within and on the surface of the vegetation or ground, and the reflection, absorption, and transmission by the atmosphere, clouds, and atmospheric contaminants [77]. The quality of the information regarding primary productivity variation encompassed in NDVI values is therefore a function of the type of processing applied on raw data [56], [78], as well as the spatial location: the relationship between NDVI and vegetation can for example be biased in low vegetated areas and very dense canopies [79]. Also, NDVI cannot distinguish between different species of plant [33]. Yet herbivores have different assortments of preferred food species [80]. Therefore NDVI may be too general to be an accurate index of resource availability for all the individual ungulate populations considered. Although limitations exist for NDVI to capture the dynamics of resource availability, remote sensing based indices remain the only possibility to obtain direct, quantified measures of this parameter at such spatial and temporal scales [33], [81].

Our expectation regarding the role of diet type in explaining breeding synchrony (Table 1) was not supported, as we were not able to report any significant effect of diet type on birth season length (all P>0.70; Table 2). The reliability of this reported result should be further assessed as more data on reproductive synchrony become available: in our dataset, only eleven of the 70 populations were classified as browsers, and most of these were located at relatively high latitudes. Contrary to expectations (Table 1), also, calf behaviour and the level of gregariousness had no effect on birth season length (all P>0.11). Although our results provide no evidence for the predator avoidance hypothesis, we cannot exclude potential effects of predator avoidance on birth synchrony. While species with following young may benefit when young are born at the same time through predator saturation and/or, confusion, it is also possible that species with hiding young benefit from the dilution effect associated with breeding synchronously [82]. If this is correct, it would explain the absence of significant effect of calf behaviour on birth season length. Another possibility is that this difference in the results confirms the suggestion that assuming independence of interspecific data can lead to biased results [35]. Predator avoidance may indeed act to shorten birth seasons of species with following young in locations with high levels of predation [83]–[85], but might not be imperative in explaining the variation in birth season length at a global scale. In general, the results of past studies which used interspecific data but did not correct for phylogenetic non-independence should be treated with caution and where possible re-analyzed.

The aim of our work was to shed light on the factors associated with a high degree of breeding synchrony in ungulates, thereby revisiting the results from the last comprehensive review on the issue [24]. Overall, both the degree of seasonal fluctuation in resource dynamics and inter-annual changes in resource availability, rather than solely the degree of seasonal fluctuation in primary productivity dynamics, was shown to shape variation in birth season length of ungulates, expanding the ideas derived from localized studies [86], [87] to a global scale. Research is required into how climate change is affecting resource availability dynamics in order to be able to tell if there will be long term negative fitness costs for ungulate populations caused by a mismatch of parturition and the optimal resource window. Experimental approaches might also be required to further validate our correlative results. It is perhaps worrying that observations of negative fitness due to trophic mismatch caused by climate warming have already been reported in cattle (*Bos taurus*) that exhibit year round breeding [16]. As we were only able to collect information for 38 species and focused our analyses on the population level (with assumed phylogenetic distances among populations close to zero), we weren't able to quantify the relative importance of evolutionary and ecological mechanisms in determining the level of breeding synchrony in ungulates. The fact that there is significant phylogenetic signal in the data implies that breeding synchrony is to some extent phylogenetically constrained and therefore not completely plastic. But the high level of variability in birth season length among populations of the same species and the low measure of phylogenetic independence (i.e., the maximum likelihood estimate of lambda) suggest a weak phylogenetic signal. Despite this, however, the results obtained from the analysis neglecting the phylogenetic inertia substantially differed from the ones we report here. Importantly, our results highlighted that the levels of seasonality and inter-annual variability in vegetation dynamics accounted for a non-negligible portion of the variation in birth season length (adjusted R^2^ = 0.5011 for our dataset), tentatively suggesting that local adaptation may play a more dominant role than niche conservatism.

## Supporting Information

Text S1 **Measurement of constancy and contingency using the method proposed by Colwell 1974.**
(DOCX)

Figure S1 **Expected (left) and observed (right) changes in birth season length (according to the best model for birth season length) with changes in NDVI constancy (i.e., our measure of inter-annual variability in NDVI dynamics) and NDVI contingency (i.e., our measure of the strength of the seasonal pattern in NDVI dynamics).** In this figure, birth season length is on the log scale. Within our dataset (used to generate this figure), contingency ranges from 0 to 0.5 and constancy from 0 to 1.
(TIFF)
