# Supplementary material for: Reassessing the Determinants of Breeding Synchrony in Ungulates
Source: PLoS One. 2012 Jul 20;7(7):e41444. doi: 10.1371/journal.pone.0041444 (PMC3401108; doi:10.1371/journal.pone.0041444)
Supplement: Text S1 — Measurement of constancy and contingency using the method proposed by Colwell 1974. (DOCX) [file pone.0041444.s001.docx]

**Supporting Information Text S1: Measurement of constancy and contingency using the method proposed by Colwell 1974**

Measures of predictability, constancy and contingency are derived from the mathematics of information theory, more precisely from the Shannon information statistics. Imagine a frequency matrix, where there are t columns representing times within a cycle (in our case 24 as we considered 2 NDVI values per month) and s rows representing the states of the phenomenon (in our case 10 different NDVI classes; 0-0.1, 0.1-0.2, …0.9-1). Let Nij be the number of cycles for which the phenomenon (in this case the NDVI value) was in state i at time j. Define the column totals (Xj), row totals (Yi) and the grand total (Z) as

$$X_{j}=\sum_{i=1}^{s} N_{ij}$$

$$Y_{i}=\sum_{j=1}^{t} N_{ij}$$

$$Z=\sum_{j} X_{j}=\sum_{i} Y_{i}=\sum_{j} \sum_{i} N_{ij}$$

Then the uncertainty with respect to time is

$$H\left( X \right)=-\sum_{j=1}^{t} \frac{X_{j}}{Z}log\left( \frac{X_{j}}{Z} \right)$$

The uncertainty with respect to state is then

$$H\left( Y \right)=-\sum_{i=1}^{s} \frac{Y_{i}}{Z}log\left( \frac{Y_{i}}{Z} \right)$$

And the uncertainty with respect to the interaction of time and state is

$$H\left( XY \right)=-\sum_{i} \sum_{j} \frac{N_{ij}}{Z}log\left( \frac{N_{ij}}{Z} \right)$$

Predictability (P) can then be defined as

$$P=1- \frac{H\left( XY \right)-H(X)}{log(s)}$$

Constancy is maximised when all rows but one are zero, while being minimised when all row totals are equal. A measure of constancy (C) with range (0-1) is given by

$$C=1- \frac{H(Y)}{log(s)}$$

Contingency represents the degree to which time determines state, or the degree to which they are dependent on each other. An adjusted measure of contingency (M) with range (0-1) is given by

$$M= \frac{H\left( X \right)+H\left( Y \right)-H(XY)}{log(s)}$$

In this scenario, predictability (P) is simply the sum of constancy (C) and contingency (M), with P=C+M.
